# ­Reproductive strategies of two common sympatric Mediterranean sponges: *Dysidea avara* (Dictyoceratida) and *Phorbas tenacior* (Poecilosclerida)

**DOI:** 10.7717/peerj.5458

**Published:** 2018-08-09

**Authors:** Sonia de Caralt, Janina González, Xavier Turon, María J. Uriz

**Affiliations:** 1 Centre d’Estudis Avançats de Blanes, Consejo Superior de Investigaciones Científicas, Blanes, Girona, Spain; 2 GRMAR, Institut d’Ecologia Aquàtica, Universitat de Girona, Girona, Spain

**Keywords:** Reproduction, Sponges, Marine invertebrates, Larvae, Mediterranean sea

## Abstract

Despite their abundance in benthic ecosystems, life cycles and reproductive features of most sponge species remain unknown. We have studied the main reproductive features of two demosponges, *Dysidea avara* and *Phorbas tenacior,* belonging to phylogenetically distant groups: Orders Dictyoceratida and Poecilosclerida, respectively. Both sponges are abundant and share habitat in the Mediterranean rocky sublittoral. They brood parenchymella larvae with different morphology and behaviour. Sampling was conducted monthly over a two-year period in a locality where both species coexist. The two species reproduced in spring-summer, and presented species-specific reproductive features despite being subject to the same environmental conditions. *D. avara* has a shorter reproductive period than *P. tenacior*, ending before the peak of temperature in summer, while the reproductive period of *P. tenacior* lasts until beginning of autumn. Brooding larvae were present in June-July in *D. avara*, and in August-October in *P. tenacior*. Larval size, reproductive effort and number of larvae produced (measured the month with the maximum production) were significantly higher in *D. avara* than in *P. tenacior*. A higher reproductive effort and larval traits point to a more opportunistic life strategy in *D. avara* than in* P. tenacior.* A lack of overlap in the timing of larval release, as well as different reproductive traits, may reduce competition and facilitate the coexistence of these two sympatric and abundant sponges.

## Introduction

Sponges are key structural elements in marine rocky bottoms (Vacelet, 1979; Uriz, Rosell & Martin, 1992) from sublittoral habitats to the deeper continental shelf (Boury-Esnault, Pansini & Uriz, 1994), where they play a paramount role in energy transfer processes (e.g., Gili & Coma, 1998; Ribes et al., 2005; De Goeij et al., 2013). Furthermore, sponges are at the base of the animal tree of life (Feuda et al., 2017) and are therefore a key group for understanding the evolution of reproductive traits in Metazoa. However, although studies on sponge reproduction proliferated steadily in the last decades (reviewed in Ereskovsky, 2010; Lanna et al., 2018a), only a tiny fraction of the sponge species has been studied so far, and new species-specific reproductive traits, which are driving factors of the species’ distribution and abundance, are being revealed (e.g.,  Abdo, Fromont & McDonald, 2008; Piscitelli et al., 2011; Pérez-Porro, González & Uriz, 2012; Koutsouveli et al., 2017). The current gaps in the knowledge of reproductive parameters of sponges prevent generalizations about reproductive strategies across taxonomic groups, growth forms, or habitat characteristics.

Sponges present a large variety of larval types (e.g., Boury-Esnault & Rützler, 1997; Maldonado & Bergquist, 2002) with diverse swimming abilities (e.g., Mariani et al., 2006; Uriz, Turon & Mariani, 2008) that likely translate into contrasting dispersal capacities in the field and may determine genetic diversity and viability of sponge populations. Although clonal reproduction is frequent in sponges (e.g., Maldonado & Uriz, 1999; Calderón et al., 2007), it is usually combined with the release of sexually produced propagules, which accounts for the genetic variability reported for sponge populations (reviewed in Uriz & Turon, 2012; Pérez-Portela & Riesgo, 2018). Most studies on population genetics of sponges show genetically structured populations (e.g., Duran et al., 2004; Calderón et al., 2007; Whalan et al., 2008; Blanquer & Uriz, 2010; Guardiola, Frotscher & Uriz, 2012), which suggests an extremely poor larval and gamete exchange even among close populations. The reproductive traits of the species, such as larval characteristics, fecundity, and timing of larval release are crucial factors in controlling the connectivity between sponge populations.

The most important environmental parameter determining reproduction in sponges is temperature (e.g., Uriz et al., 1995; Fromont, 1999; Ereskovsky, 2000; Bautista-Guerrero, Carballo & Maldonado, 2014; Lanna et al., 2018b). However, other environmental characteristics, such as photoperiod (Abdo, Fromont & McDonald, 2008), food availability (Witte, 1996; Lanna et al., 2015; Lanna et al., 2018b; Spetland et al., 2007), hydrodynamics (Mariani, Uriz & Turon, 2005; Abdo, Fromont & McDonald, 2008; Abdul Wahab et al., 2014), lunar phase (Nozawa, Huang & Hirose, 2016), or presence of stressors (De Caralt & Cebrian, 2013) can also influence reproductive cycles and larval production in sponges. In the Mediterranean, many sponge species reproduce in spring/summer, during the warm period, albeit there are instances of species releasing larvae during autumn or even winter (Mariani, Uriz & Turon, 2005).

This study focuses on the common demosponges *Dysidea avara* (Order Dictyoceratida), and *Phorbas tenacior* (Order Poecilosclerida), which share habitat in the rocky sublittoral of the Mediterranean Sea. Both species have different growth forms: thinly encrusting in *P. tenacior* and thick sheets with protruding thick oscular chimneys in *D.  avara*. The two species have a similar Atlanto-Mediterranean distribution (Cruz, 2002; Van Soest et al., 2018).

The two species present contrasting larval characteristics (Mariani, Uriz & Turon, 2005): the larva of *D. avara* is a typical dictyoceratid parenchymella, relatively large and solid with abundant reserves and collagen bands linking the peripheral and inner layers. These features correlate with good swimming abilities and potentially long lifespan of dictyoceratid larvae (Erevskosky & Tokina, 2004; Uriz, Turon & Mariani, 2008). In addition, these larvae also have a ring of long cilia and phototactic cells at the posterior end, which determine an active directional swimming and facilitate substrate selection (Mariani, Uriz & Turon, 2005; Mariani et al., 2006). Conversely, the larva of *P. tenacior* belongs in the poecilosclerid larval type, which is smaller and with less reserves (Mariani, Uriz & Turon, 2005), indicating poorer swimming capabilities and shorter larval lifespans. Poecilosclerid-like larvae also lack the posterior ring of long cilia and show a non-directional swimming behaviour (Mariani et al., 2006; Uriz, Turon & Mariani, 2008).

This study aims at providing an accurate assessment of the reproductive cycles of these target species and their reproductive effort in terms of investment in reproductive structures and larval production. We wanted to contribute new data on reproductive strategies of sponges and to assess whether these can facilitate the coexistence of two abundant species.

## Materials and Methods

### Sampling procedure and temperature monitoring

**Figure 1:**
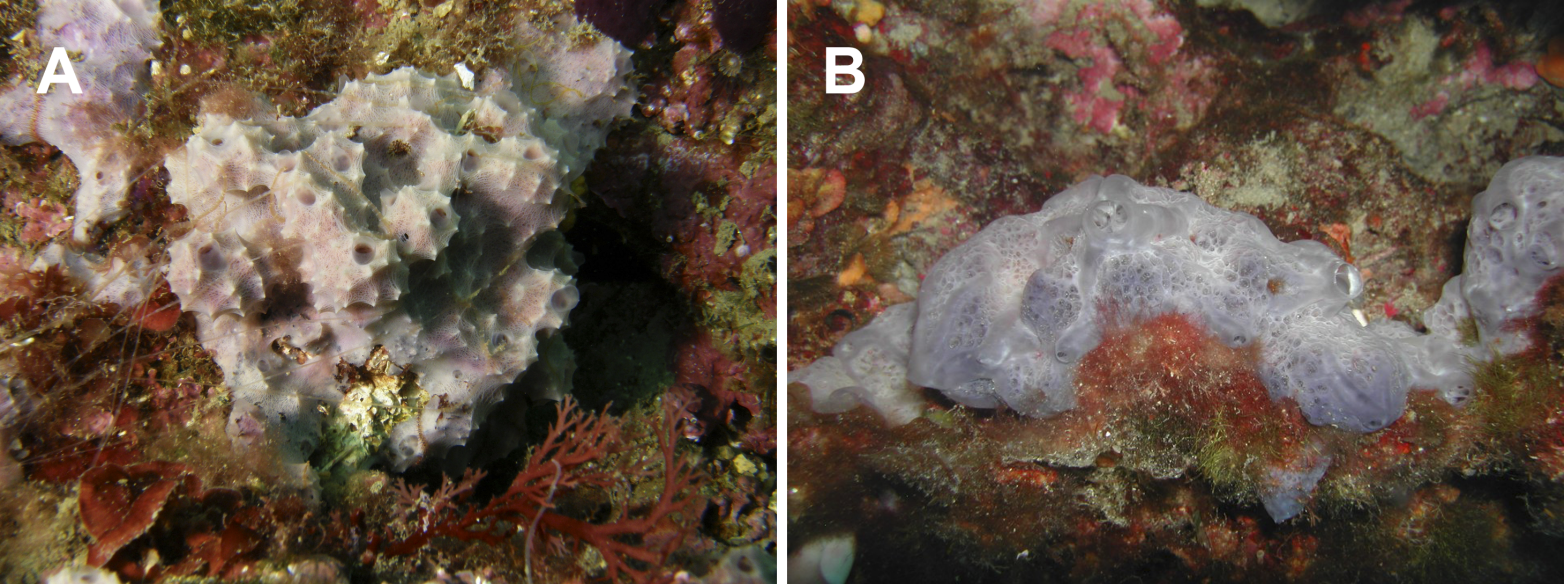
Field pictures of the two species at the study site. (A) *D. avara;* (B) *P. tenacior*. Images by J  González

Samples of *D. avara* and *P. tenacior* (Fig. 1) were collected monthly in the locality of l’Escala (42°06′52″N, 3°10′07″E) in the North-western Mediterranean Sea, from March 2009 to March 2011. The sampling was not destructive, and the approval of the funding by the Spanish Government (project CTM2007-66635) includes the permission to perform the sampling activities foreseen in the working plan in Spanish waters. All samples were collected by SCUBA diving from a population sitting on a long rocky wall facing NW, between 10 and 14 m in depth. Individuals of size larger than ca. 50 cm^2^ were haphazardly selected, and from 20 to 30 individuals per species (25–30 in most cases) were sampled monthly. To avoid sampling the same individuals in subsequent months, we selected different subareas of the rocky wall each month. Sampling was minimally invasive, as only a fragment of ca. 1 cm^2^ was taken from each individual

Temperature was recorded *in situ* at hourly intervals using a Stowaway Tidbits^®^, autonomous data logger (0.2 °C precision), placed at the study site at 14 m of depth. Photoperiod data were obtained from the US Naval Observatory (http://aa.usno.navy.mil).

### Histological sections

Samples were fixed immediately in a solution of 5% formaldehyde in seawater. Prior to the histological procedures, the samples of *P. tenacior*, which contained siliceous spicules, were desilicified for 2 h in a solution of 5% hydrofluoric acid, while *D. avara* samples were decalcified for 2 h in a 5% solution of ethylene-diamine-tetracetic acid (EDTA) (Eerkes-Medrano & Leys, 2006) to remove the calcareous material included in its protein-made skeletal fibers (Galera et al., 2000).

Samples of both species were subsequently rinsed in distilled water, dehydrated through a graded ethanol series (70%, 96%, and absolute), rinsed in toluene/ethanol (1/1), and then in pure toluene, and embedded in paraffin for histological examination. Histological sections, 5 µm-thick, were obtained using an Autocut Reichert-Jung microtome 2040 (R. Jung GmbH, Nubloch, Germany). Sections were deparaffined with xylene, stained with hematoxylin and examined through a Zeiss Axioplan II compound microscope connected to a digital camera (Prog Res™ C 10^plus^ from JENOPTIK). Four sections were cut from each individual sponge, separated at intervals of ca. 1 mm to avoid cutting more than once the same reproductive structures.

### Reproductive traits

Digital images were used to count and measure the diameter and area of the reproductive elements (i.e., spermatocysts, oocytes, embryos, and larvae), which were manually outlined and measured using Prog Res CapturePro v2.8.0 software. Five different zones, randomly chosen from each sponge section, were acquired. The area of the observation field was determined to calculate the reproductive effort per surface unit. A total area of ca. 8 mm^2^ was examined per sponge individual (4 sections ×  5 random fields of ca. 0.4 mm^2^).

The following variables were considered: (1) percentage of individuals in reproduction (i.e., containing any reproductive element), (2) mean diameter of spermatocysts, oocytes, embryos and larvae (measured as the longest dimension of the corresponding element), (3) reproductive effort measured as both number of reproductive elements per surface area and relative area of the sponge sections occupied by those elements, and (4) monthly maximum number of offspring (embryos and larvae) found in sponge sections. This maximum occurs always in one of the last two months of reproductive activity. We acknowledge that this measure provides a conservative estimate, as some larvae could have been released before the observation. However, the alternative approach of summing embryos and larvae over different observation times would likely result in a gross overestimation.

### Statistical analyses

Differences in mean larval sizes (pooling years) between the sponge species were analysed by *t*-tests. Differences in percent of individuals in reproduction, reproductive effort, and number of offspring were compared by two-way ANOVAs with species and year as fixed factors. For the percent of individuals in reproduction we used as replicates the months of reproductive activity. For the reproductive effort (in relative area) the replicates were the individuals in the month with the highest effort of each year. Finally, for the number of offspring we used as replicates the individuals in the month with the highest number of embryos plus larvae. Normality and homogeneity of variances were examined by Kolmogorov–Smirnov and Levene tests, respectively. For one variable, log transformation was necessary to comply with these assumptions (see ‘Results’).

The time course of reproductive effort (in relative area) of both species was correlated with the seawater temperature and with photoperiod using cross-correlation analyses. In these, relationships between two time-series are analysed by lagging one series with respect to the other. Correlation at time lag 0 is the usual Pearson correlation, correlations at negative time lags relate values of the first series to previous values in the second series, and the reverse is true for positive time lags.

Analyses were done with STATISTICA v6 (StatSoft, Inc., Tulsa, OK, USA) and SYSTAT v12 (Systat Software, Inc., San Jose, CA, USA). Graphs were plotted with SigmaPlot v10 (Systat Software, Inc., San Jose, CA, USA) and the R package ggplot2 (Wickham, 2009). The raw data corresponding to the different variables measured are presented in Table S1.

## Results

### Reproductive cycle

**Figure 2:**
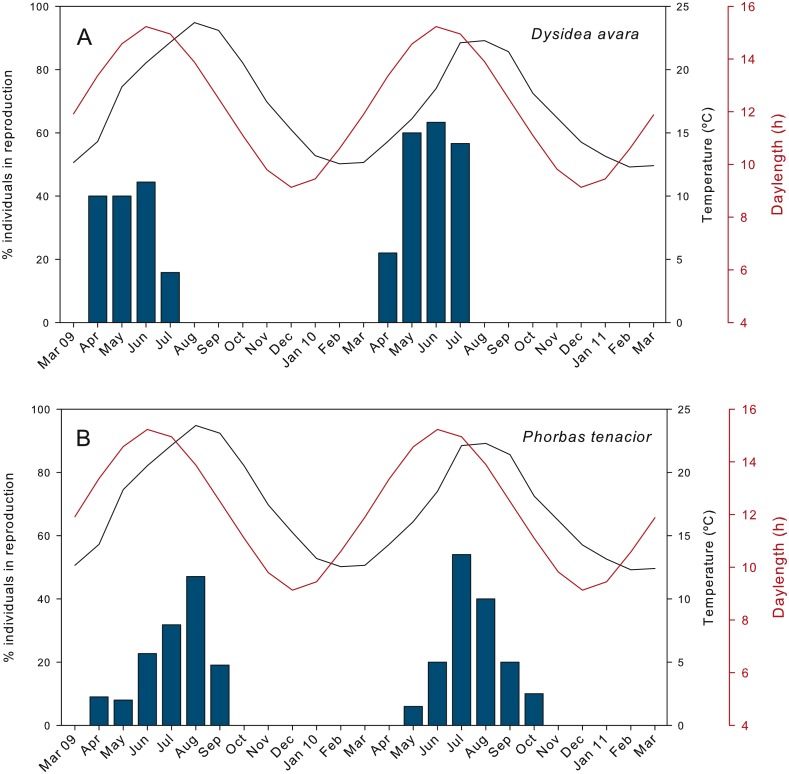
Percentage of individuals in reproduction during the two monitored years. (A) *D. avara*; (B) *P. tenacior*. The temperature and daylenght graphs are indicated.

The percentage of actively reproducing individuals of *D. avara* reached maximum values in June in both study years, but this percentage varied between years (ca. 44% in 2009 and 63% in 2010) (Fig. 2A). In *P. tenacior* this percentage reached maximum values in August 2009 (47%) and in July 2010 (ca. 54%) (Fig. 2B). The percentage of individuals in reproduction was significantly higher in *D. avara* than in *P. tenacior* (mean ± SE: 42.66 ± 5.7 vs 25.87 ± 4.9 over the reproductive period of each species), while the effect of year or the interaction between year and species were not significant (two-way ANOVA, Table 1A).

**Figure 3:**
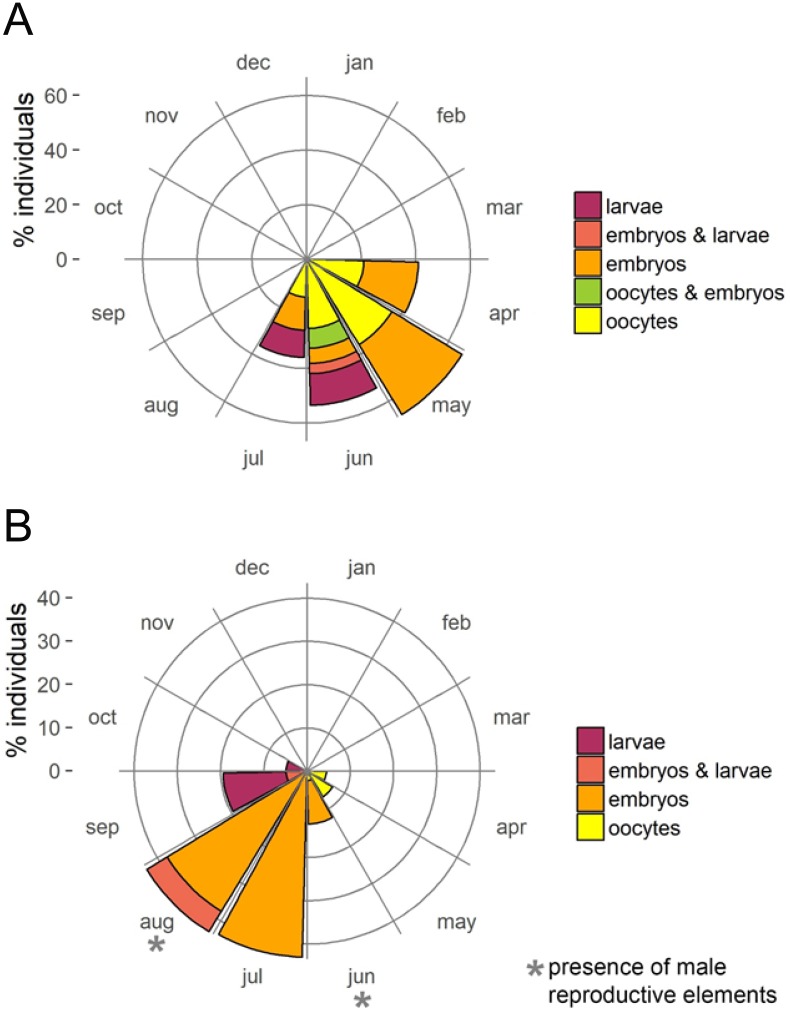
Circular plots of percent individuals with the different reproductive structures averaged over the two study years. (A) *D. avara*; (B) *P. tenacior*

**Figure 4:**
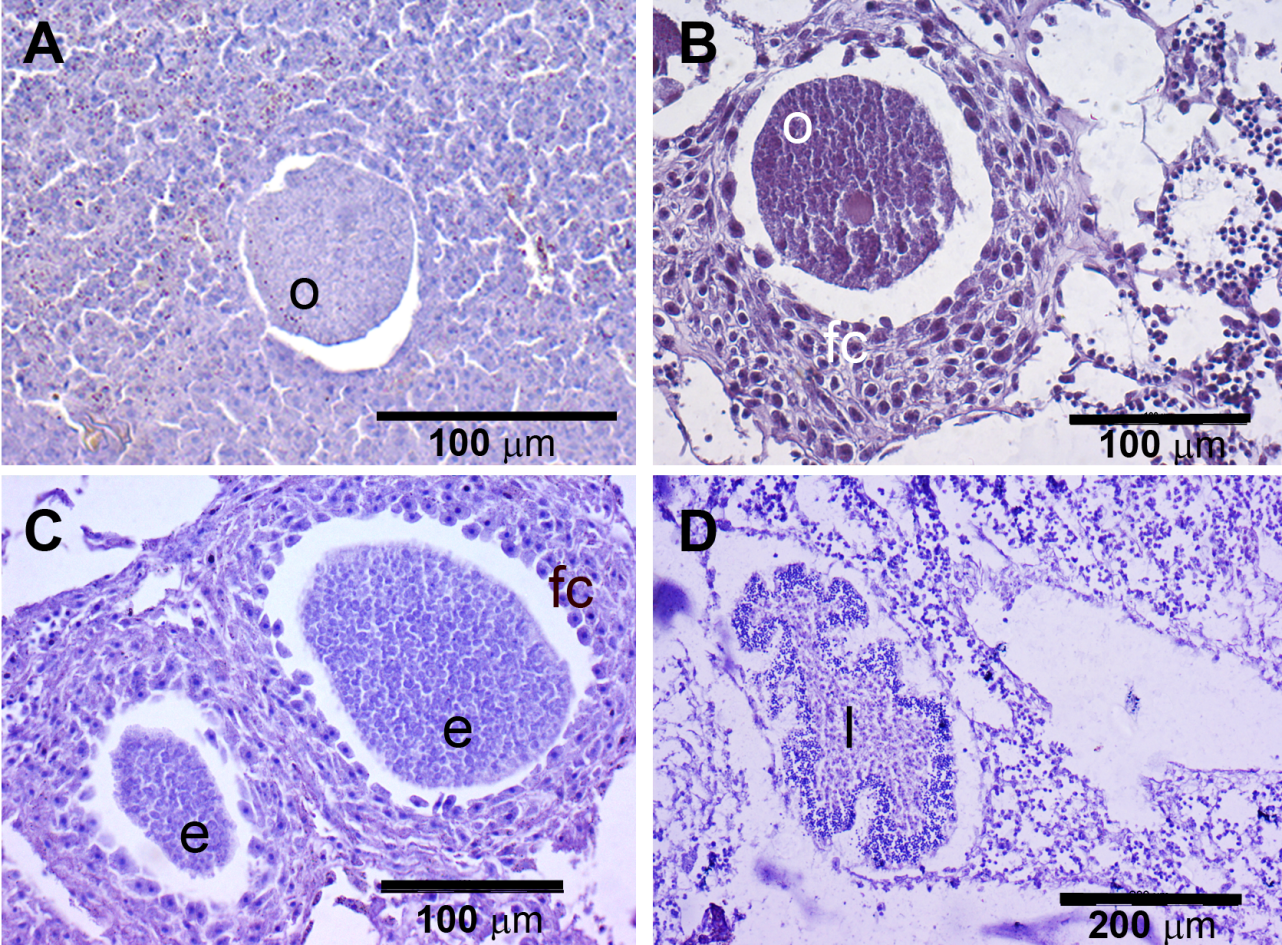
Histological sections of *D. avara.* (A) Immature oocyte (o); (B) mature oocyte (o), and follicular cells (fc); (C) embryos (e), and follicular cells (fc); (D) larva (l) with a wrinkled appearance.

**Figure 5:**
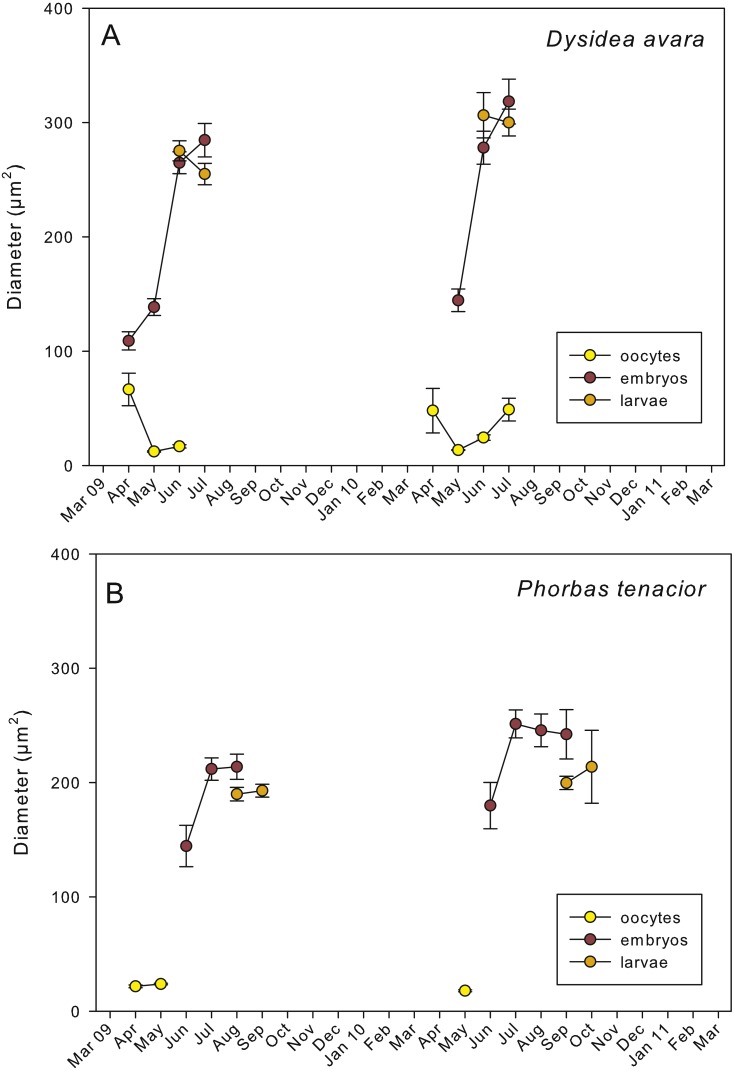
Mean diameter of the oocytes, embryos, and larvae. (A) *D. avara;* (B) *P. tenacior*. Vertical bars are standard errors.

The presence of reproductive elements (Fig. 2A) indicated that the reproductive period of *D. avara* lasted for four months (April to July) each year. The beginning of the reproductive period coincided with temperatures above 14 °C. No spermatocysts were found in the two years sampled. Thus, the hermaphroditic or gonochoric character of this species could not be ascertained. The detection of individuals with the different reproductive structures (Fig.  3A, years averaged) showed that oocytes and embryos can be present during the whole reproductive period, while larvae appeared the last two months. Some individuals harboured at the same time oocytes and embryos, or embryos and larvae, but the three stages were never detected at the same time in a given individual (Fig. 3A). Oogenesis lasted from April to June 2009 and from April to July 2010. Over these periods, oocytes changed from spherical to elliptical in shape with a large nucleolated nucleus (Figs. 4A and 4B). Oocytes were surrounded by a layer of polygonal follicular cells with the nucleus in a central location (Fig. 4B). Oogenesis was asynchronous within and between individuals, with different stages commonly found in the same sponge section. The mean diameter of the oocytes varied between months (Fig. 5A), with maximum values of 66.6 ± 14.2μm (mean ± SE) in April 2009 and minimum of 12.22 ± 0.5μm in May 2009. In both years, there was a marked decrease of oocyte size from the first month of observation (April) to the second, with a progressive increase afterwards (Fig. 5A). This suggests that an initial batch of oocytes turned quickly into embryos, and new oocytes were generated and started to grow in the following months.

**Table 1:** Two-way ANOVA results. (A) Percentage of individuals in reproduction; (B) reproductive effort; (C) maximum number of offspring, with species and year as fixed factors.

Source of variation	*DF*	SS	MS	*F*	*P*
**A. Percentage of individuals in reproduction**					
year	1	532.869	532.869	1.307	0.261
species	1	1,301.871	1,301.871	15.029	<0.001
year × species	1	109.913	109.913	0.321	0.574
residual	15	3,980.176	265.345		
**B. Investment in reproduction**					
year	1	67.283	67.283	1.281	0.265
species	1	768.016	768.016	14.630	<0.001
year × species	1	15.976	15.976	0.304	0.585
residual	34	1,784.759	52.493		
**C. Maximum number of offspring^a^**					
year	1	0.004	0.004	0.032	0.858
species	1	0.701	0.701	5.428	0.026
year × species	1	0.298	0.298	2.309	0.138
residual	34	4.392	0.129		

**Notes.**

a Log transformed data.

Embryogenesis of *D. avara* occurred throughout the sponge mesohyl. Embryos were first observed in April of the first study year and in May of the second year and were present within the sponge tissues until the last month of reproductive activity. Embryo development was asynchronous within individuals, as mature and immature embryos coexisted in the same individual. A sheet of follicular cells surrounded embryos (Fig. 4C). Mean diameter of embryos increased with time in both years, ranging from 109.03 ± 7.9μm (mean ± SE) in April 2009 and 144.50 ± 9.9μm in May 2010 to 284.64 ± 14.6 and 318.42 ± 19.6μm in July 2009 and 2010, respectively (Fig. 5A).

The parenchymella larvae of *D. avara*, similar in size to mature embryos, showed a distinct external layer of elongated cells (Fig. 4D). Larvae were first observed in June and remained in the sponge mesohyl for two months (both years), during which they decreased slightly in size (Fig. 5A) likely due to contraction, as they adopted a wrinkled appearance (Fig. 4D). Their sizes ranged from 275.32 ± 8.8μm (June 2009) to 255 ±9.35μm (July 2009) and from 306.43 ± 19.8μm (June 2010) to 300.04 ± 11.7μm (July 2010) (Fig. 5A). Larval production started with temperatures above 20 °C (2009) and 18 °C (2010), and spawning was over before temperatures reached their maxima in August.

**Figure 6:**
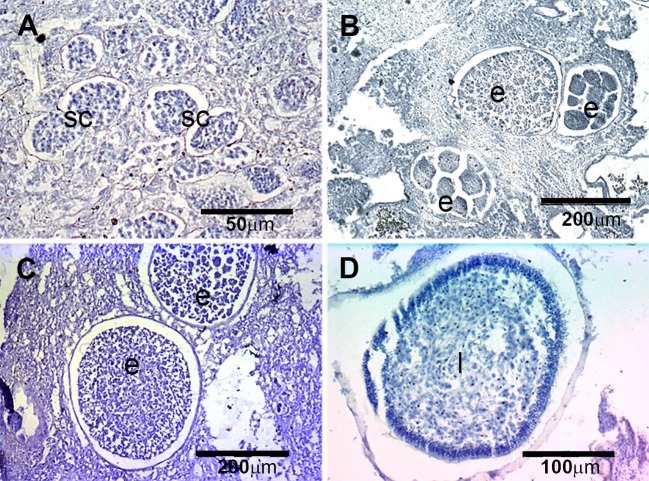
Histological sections of *P. tenacior.* (A) Spermatic cyst (sc); (B–C) several stages of embryo development (e); (D) larva (l).

The reproductive period of *P. tenacior* extended over six months, starting in April (2009) or May (2010) (Fig. 2B). Reproduction was triggered when temperatures were above 14 °C in 2009 and above 16 °C in 2010. Spermatogenesis was a punctual event, only recorded in June 2009 and August 2010. The species is hermaphroditic, since in all cases, individuals with spermatic cysts had also developing embryos. The spermatic cysts observed were all at the same stage of development and were spherical in shape (Fig. 6A), with a uniform mean diameter of 21.96 ± 0.8μm (mean ± SE, both years pooled).

Oogenesis, embryogenesis, and larval development periods were sequential in *P. tenacior*, with little temporal overlap of these stages. Pooling both years (Fig. 3B), oocytes were present during the first part of the reproductive cycle only (April–June), while embryos were first detected in June and remained until September. The embryos and larvae in *P.  tenacior* were found throughout the sponge mesohyl. Larvae were found between August and October, but were only present during two months in a given year. Larval production and spawning took place when the temperatures reached their maxima (ca. 23 and 22 °C in 2009 and 2010, respectively).

Spherical oocytes showed similar diameters with an average of 21.58 ± 0.7μm, mean ± SE for both monitored years (Fig. 5B). Several stages of embryo development were simultaneously present in the same individual. Embryo mean diameter increased with time, reaching its maximum in August 2009 and July 2010 (213.82 ± 11.0 and 251.29 ± 12.2μm, respectively) (Figs. 5B, 6B and 6C). Larvae showed the characteristic external layer of ciliated cells (Fig. 6D) and measured up to 192.89 ± 5.6μm in 2009 and 213.80 ± 31.9μm in 2010. Their size remained approximately constant during the two incubation months (Fig. 5B). Overall, larvae of *P. tenacior* were significantly smaller than those of *D. avara* (*p* < 0.001, *t*-test).

### Reproductive effort

**Figure 7:**
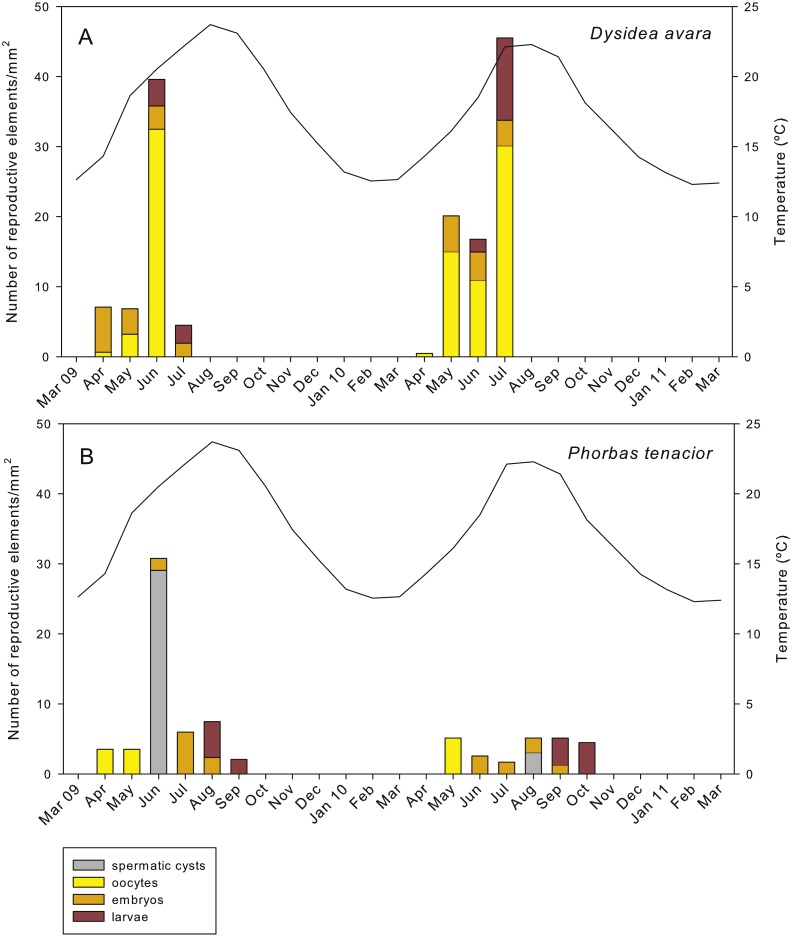
Abundance of the different reproductive structures per unit area in sponge sections during the study period. (A) *D. avara*; (B) *P. tenacior*. The temperature graph is indicated

**Figure 8:**
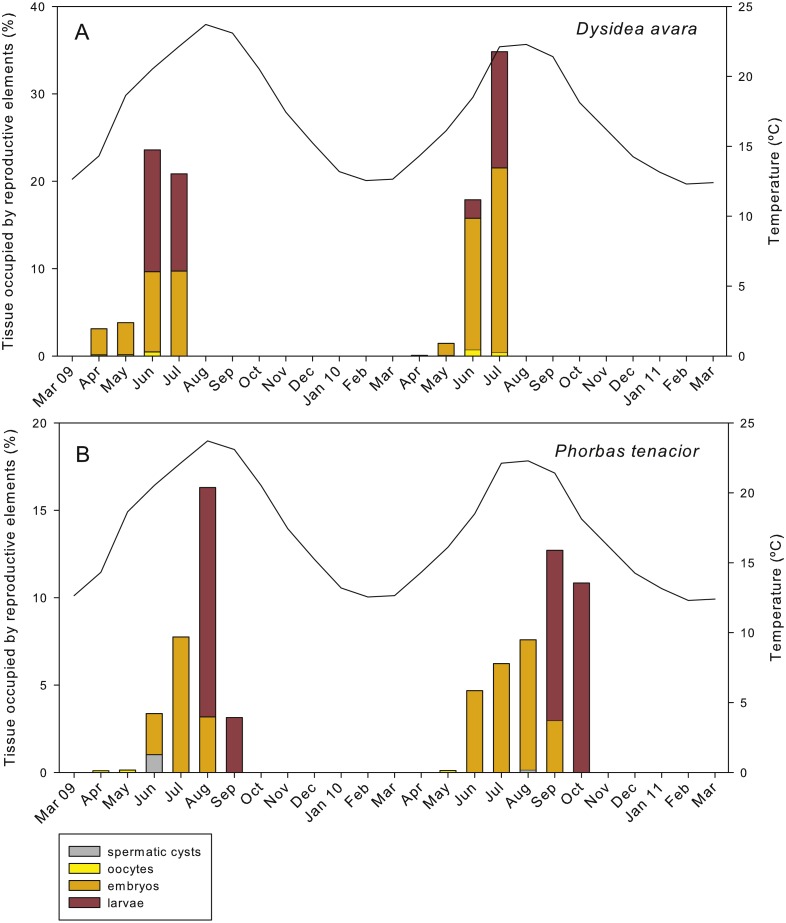
Relative area occupied by the different reproductive structures in sponge sections during the study period. (A) *D. avara*; (B) *P. tenacior*. The temperature graph is indicated

In *D. avara*, oocytes were the most abundant reproductive element. In terms of abundance, oocyte density varied with time being highest in June 2009 (32.48 ± 20.4 oocytes/mm^2^, mean ± SE) and in July 2010 (30.13 ± 7.1 oocytes/mm^2^) (Fig. 7A). In terms of area, however, oocytes occupied only a tiny fraction of the sponge mesohyl, between 0.07 and 0.73% (Fig. 8A).

Embryos showed similar abundance with time in both years, ranging from ca. 2 to 7 embryos/mm^2^, with the maxima in April 2009 (6.44 ± 1.4, mean ± SE) and May 2010 (5.13 ± 0.9) (Fig. 7A). The percentage of tissue occupied by embryos was highly variable among individuals and increased with time in both monitored years, reaching mean values of ca. 10% and 20% in July 2009 and 2010, respectively (Fig. 8A). Larval abundance per mm^2^ of sponge tissue ranged from 1.82 ± 0.1 in June 2010 to the maximum recorded in July 2010 (11.75 ± 3.6) (Fig. 7A). The percentage of tissue occupied by larvae was lowest in June 2010 (2.10 ± 1.0) and highest in June 2009 (13.94 ± 1.3, Fig. 8A).

In *P. tenacior*, spermatic cysts were recorded in June 2009 at densities of 29.06 ± 7.3 spermatic cysts/mm^2^, mean ± SE) and in August 2010 at lower densities (3.04 ±1.3) (Fig. 7B). Spermatic cysts occupied a low percentage of the sponge tissue (1.02% ±0.4 in June 2009 and 0.15% ± 0.1 in August 2010) (Fig. 8B).

The abundance of oocytes in *P. tenacior* ranged from 3.53 ± 0.3 to 5.13 ± 2.6 oocytes/mm^2^ (mean ± SE) (Fig. 7B). They represented always less than 0.15% of the sponge tissue (Fig. 8B). The embryo density per mm^2^ was from 1.68 ± 0.5 to 5.98 ± 0.6 embryos/mm^2^ (Fig. 7B), with embryos occupying from ca. 2% to 8% of the sponge tissue (Fig. 8B). Larval density decreased from 5.13 ± 2.6 (August) to 2.08 ± 0.6 larvae/mm^2^ (September) in 2009 while differences were less marked in 2010, ranging from 3.84 ± 0.7 (September) to 4.48 ± 0.6 (October) (Fig. 7B). Likewise, area of tissue occupied by larvae decreased in 2009 from August (13.12% of sponge tissue) to September (3.14%) while in 2010 it did not change appreciably (9.73% in September, 10.84% in October, Fig. 8B).

The total reproductive effort in area in *D. avara,* including oocytes, embryos and larvae, reached its maximum in June 2009 and July 2010, with the reproductive tissue accounting for 23.59% and 34.83%, respectively, of the total sponge tissue (Fig. 8A). In *P. tenacior* total reproductive effort peaked in August 2009 and September 2010, with the reproductive tissue representing 16.3% and 12.71%, respectively, of the total sponge tissue (Fig. 8B). Comparing the months with the maximum reproductive effort in both species, this value was significantly higher (two-way ANOVA) in *D. avara* than in *P. tenacior*, while year or the interaction of species with year did not have a significant effect (Table 1B).

The reproductive effort (in area) of *D. avara* was significantly correlated with temperature (Fig. S1A), but the correlation was higher with the temperature in the following two months (time lags of +1 and +2), indicating an advancement of reproduction in this species with respect to temperature maxima. Conversely, the reproductive effort of *P. tenacior* had the highest correlation with temperature in the current month (time lag 0) (Fig.  S1B), indicating a close coupling of the time course of both variables. The picture changes when considering photoperiod (measured as day length), which had the highest correlation with reproductive effort in the current month in *D. avara*, while for *P. tenacior* the correlation was maximal with day length in the previous months (time lags of −1 and  −2).

### Maximum number of offspring

Considering the month with the highest abundance of embryos plus larvae each year this abundance was variable between years in *D. avara* (3.93 ± 0.6 embryos plus larvae/mm^2^ in June 2009 versus 8.06 ± 2.3 embryos plus larvae/mm^2^ in July 2010, mean ± SE). In *P. tenacior* differences between years were also noticeable (2.90 ± 0.6 embryos plus larvae/mm^2^ versus 4.49 ± 0.6 larvae/mm^2^ in August 2009 and September 2010, respectively, Figs. 7A and 7B). On average, *D. avara* produced ca. 1.66 more propagules per unit area than *P. tenacior*, being the differences statistically significant between species, but not between years or when testing the interaction of species and years (two-way ANOVA, Table 1C).

## Discussion

The study of the reproductive cycle of *D. avara* and *P. tenacior* over two consecutive years revealed seasonal cycles with some important differences between species. In both cases, reproduction occurred in spring-summer and the first reproductive structures appeared in April-May. Both sponges reproduced seasonally, as commonly reported for sponge species from temperate regions (Lanna et al., 2018b). However, the reproductive cycle of *D. avara* lasted only for four months and reproduction was over before water temperature reached its maximum in August. Other Dictyoceratida species have been reported to contain oocytes all year round, but with embryogenesis restricted to 4 to 8 months, depending on the species (Zarrouk et al., 2013; Mercurio et al., 2013; Baldacconi et al., 2007; Lanna et al., 2018b). The reproductive period of *P. tenacior* spanned over six months, ending at the beginning of fall. This timing is similar to other Poecilosclerida species (Ilan, 1995; Corriero et al., 1998; Ereskovsky, 2000; Lanna et al., 2018b). Overall, both study species showed a constrained reproductive period, in accordance with most temperate sponges. It is noteworthy, however, than even during the months with the highest reproductive activity, the percentage of sponges not engaged in reproduction was of the order of 40% (*D. avara*) and 50% (*P. tenacior*). Similar ratios were found in other Dictyoceratida (Zarrouk et al., 2013; Lanna et al., 2018b) and Poecilosclerida species (Lanna et al., 2018b).

In *D. avara,* developing oocytes and embryos coexisted over the whole reproductive period, while in *P. tenacior* the different developmental stages (oocytes, embryos, and larvae) appeared sequentially, with little temporal overlap. The reproductive effort of both studied sponges is in the range of other phylogenetically related species (Dictyoceratida: Whalan, Battershill & De Nys, 2007; Zarrouk et al., 2013; Mercurio et al., 2013; Poecilosclerida: Corriero et al., 1998; Ereskovsky, 2000).

The peak of reproductive activity, both in terms of percentage of individuals and reproductive effort in *P. tenacior* coincided with the warmest temperatures. In *D. avara*, the reproductive effort peaked one to two months in advance of the summer temperature maximum. This is also reflected in the cross-correlation analyses, with highest correlation at time lag 0 for *P. tenacior*, and at time lags of +1 and +2 for *D. avara*. However, both species have a similar Atlanto-Mediterranean distribution (Cruz, 2002; Van Soest et al., 2018). The reproduction timing and growth of Atlanto-Mediterranean sponges in the Mediterranean is often correlated with their geographic origin (Blanquer, Uriz & Agell, 2008; Garate, Blanquer & Uriz, 2017) with presumably Atlantic species not reproducing in summer, the period of highest temperatures and trophic depletion (Turon, 1988; Coma et al., 2000). The reproductive timings of the two study species suggest an Atlantic origin for *D. avara*, and a Mediterranean origin for *P. tenacior*, which should be confirmed by studies of population genetics.

Other environmental variables can also influence the reproductive traits. Photoperiod, for instance, may act as a trigger for the onset of reproduction (Abdo, Fromont & McDonald, 2008). In our case, however, the start or end of the reproductive periods did not coincide with peaks in photoperiod. The close coupling of photoperiod with temperature cycles makes it difficult to tell apart the effect of both variables. Food availability is another factor that can determine the timing of reproduction in sponges (Witte, 1996), both fuelling reproductive effort and providing settlers with favourable conditions. However, in the NW Mediterranean, phytoplankton blooms occur in late winter (López et al., 1998; Cebrián & Valella, 1999), well out of the reproductive periods detected, so food availability might not be a relevant factor in our case. Alternatively, the differences found may not be the result of environmental variables, but may simply reflect the phylogenetic distance of the two species (different Orders) and their reproductive features may be phylogenetically constrained. A recent work also reported contrasting reproductive parameters in sponges belonging to different Orders (Abdul Wahab et al., 2016).

The factor year was not significant for the different parameters analysed, nor was the interaction of year with species. So, even if there were some differences in reproductive traits, we could not substantiate any inter-annual trend. This is in stark contrast with results reported for other sponge species (e.g., Bergquist, 1978; Corriero et al., 1998; Mercurio, Corriero & Gaino, 2007; Riesgo & Maldonado, 2008; Piscitelli et al., 2011; Huang et al., 2016). We can point out here that temperature minima were similar in the two years studied, while the maxima were higher in 2009, albeit slightly so (ca 1.5 °C).

Long spermatogenesis periods, spanning several months, have been reported in tropical sponges (Ettinger-Epstein et al., 2007; Whalan, Battershill & De Nys, 2007; Abdul Wahab, De Nys & Whalan, 2012), likely with repeated short sperm release events (Ilan, 1995). However, spermatogenesis is generally completed in a short time in temperate sponges (Scalera-Liaci, Sciscioli & Materrese, 1973; Corriero et al., 1998; Lepore et al., 2000; Riesgo & Maldonado, 2008; Piscitelli et al., 2011; Ereskovsky, Geronimo & Perez, 2017). A single short period of spermatogenesis can explain why we detected male structures only in two sampling months (June 2009 and August 2010) in *P. tenacior* and none in *D. avara*. When detected (*P. tenacior)*, spermatic cysts were present at high densities (e.g., in June 2009) as reported for other sponge species (e.g. Piscitelli et al., 2011; Mercurio et al., 2013; Abdul Wahab, De Nys & Whalan, 2012) and occupied similar percentages of tissue than in other Poecilosclerid species (Pérez-Porro, González & Uriz, 2012). As the presence of embryos requires previous oocyte fertilization, we can assume that spermatogenesis already occurred in April 2009 and May 2010 (before the first embryo recording) in *D. avara*. A finer sampling in time (e.g., every week), ideally performed on the same specimens, would be necessary to adequately monitor spermatogenesis in the studied sponges, and to determine the hermaphroditic or gonochoric nature of *D. avara*.

Larvae were present in the sponge tissues the last two months of the reproductive cycle in both species. The number of larvae decreased from the penultimate month to the last one (except for *D. avara* in 2010), indicating a gradual release. This is a common strategy of brooding sponges, which may increase the chances of finding favourable settling conditions (e.g. Ilan & Loya, 1990; Baldacconi et al., 2007; De Caralt et al., 2007; Pérez-Porro, González & Uriz, 2012). It is interesting to note that the period of larval brooding in the two species did not overlap, so larvae of these two abundant species would not be competing for settlement places, which may be a crucial factor in space-saturated habitats such as the sublittoral community studied here.

The percentage of individuals involved in reproduction, the effort in reproductive elements and the reproductive output were significantly higher in *D. avara* than in *P. tenacior.* Moreover, *D. avara* larvae are significantly larger and they are efficient swimmers, equipped with a posterior tuft of long cilia, while the larvae of *P. tenacior* are smaller, poor swimmers, and lack the posterior tuft of cilia (Mariani et al., 2006). The better dispersal abilities and the higher number of larvae (measured the month with the maximum production) of *D. avara* with respect to *P. tenacior* point to a more opportunistic strategy of the former species. The staggering of the reproductive timing, with no overlap of larval release periods, coupled with different reproductive traits and larval dispersal capabilities may reduce competition and favour the coexistence of these sympatric sponge species.

## Conclusions

The two sponges studied had a seasonal reproductive period in spring-summer, but its duration was longer (6 months) in *P. tenacior* than in *D. avara* (4 months).

Reproductive effort in *P. tenacior* was maximal in the period of highest temperatures, while in *D. avara* the reproductive effort peaked one to two months before the maximum of temperature.

The percentage of individuals involved in reproduction, the investment in reproductive elements and the number of offspring produced were significantly higher in *D. avara* than in *P. tenacior.* Coupled with larval features, these parameters suggest a more opportunistic strategy of the former.

Studies comparing reproductive cycles of sponge species can help to understand the biological strategies in this group. In our case, the analysis of reproductive traits in two sponges reveals significant differences, pointing to different life strategies and limited competition in sponges sharing habitat.

##  Supplemental Information

10.7717/peerj.5458/supp-1 Figure S1 Cross correlation plots. The correlation of total reproductive investment (as sum of the relative areas of reproductive structures) with temperature is indicated at time lags of one month (A) *Dysidea avara*; (B) *Phorbas tenacior*. The red lines indicate the boundaries of significant (at *p* = 0.05) correlation coefficient values.

10.7717/peerj.5458/supp-2 Figure S2 Cross correlation plots. The correlation of total reproductive effort (as sum of the relative areas of reproductive structures) with photoperiod (measured as daylength) is indicated at time lags of one month (A) *Dysidea avara*; (B) *Phorbas tenacior*. The red lines indicate the boundaries of significant (at *p* = 0.05) correlation coefficient values.

10.7717/peerj.5458/supp-3 Table S1 Source data of the different variables measured in the two sponge species Percentage of individuals in reproduction (%), Relative area of the sponge sections occupied by reproductive elements (%), number of reproductive elements per surface area (number/mm^2^), diameter of reproductive elements (µm).

## Additional Information and Declarations

### Field Study Permissions

The following information was supplied relating to field study approvals (i.e., approving body and any reference numbers): The sampling was not destructive, and the approval of the funding by the Spanish Government (project CTM2007-66635) includes the permission to perform the sampling activities foreseen in the working plan in Spanish waters.

### Data Availability

The following information was supplied regarding data availability: The raw data are provided in Table S1.
